# The efficacy of silver needle therapy for treating low back pain: a protocol for meta-analysis of randomized controlled trials

**DOI:** 10.3389/fmed.2024.1355262

**Published:** 2024-04-02

**Authors:** Wangyu Li, Xueru Xu, Rongguo Liu

**Affiliations:** Department of Pain Management, Fujian Provincial Hospital, Shengli Clinical Medical College of Fujian Medical University, Fuzhou, Fujian, China

**Keywords:** silver needle therapy, low back pain, meta-analysis, protocol, randomized controlled trials

## Abstract

**Background:**

As population aging and unhealthy living habits may exacerbate the prevalence and burden of low back pain (LBP), effective treatment and improvement of patient quality of life are particularly critical. Silver needle therapy (SNT), having evolved from traditional acupuncture, involves placing silver needles into muscles, tendons, and fascia for treatment. However, it still lacks robust clinical evidence to substantiate its effectiveness. Therefore, it is necessary to conduct more emphasis on meta-analysis to evaluate the clinical efficacy of SNT for treating LBP.

**Methods:**

We will search PubMed, Medline, Cochrane Library, Embase, China National Knowledge Infrastructure (CNKI), and Wanfang Databases up until December 2023 to identify randomized controlled trials of SNT treatment in adult patients with LBP. The primary outcome will be the intensity of pain after pain management. Secondary outcomes will include the Oswestry Disability Index, Japanese Orthopedic Association Back Pain Evaluation Questionnaire, requirement for analgesic drugs, and treatment-related adverse reactions. Two investigators conducted the literature search, selected studies that might meet the inclusion criteria based on the title and abstract, and extracted data from the eligible literature independently and will independently assess the risk of bias using the Revised Cochrane Risk-of-Bias (RoB2) tool. Multivariate analyses (including subgroup analysis, trial sequential analysis (TSA), sensitivity analysis, etc.) will be conducted to improve the quality of evidence.

**Clinical trial registration:**

Registration: PROSPERO Registration Number: CRD42023466207, https://www.crd.york.ac.uk/prospero/display_record.php?ID=CRD42023466207.

## 1 Introduction

Low back pain (LBP) is characterized by pain, stiffness, or muscle tension, with pathological changes in muscles, fasciae, and ligaments being one of the significant causes. It typically occurs between the lower rib margin and the buttock crease, with or without associated leg pain and symptoms of the lower limb nervous system (1, 2). LBP is a prevalent condition worldwide, with 568.4 million cases globally, and the incidence increases with age (3). A study assessing years lived with disability for 354 diseases across 195 countries/regions found that LBP is the leading cause of disability-adjusted life years and productivity loss globally, across 126 countries/regions (4). As population aging and unhealthy living habits may exacerbate the prevalence and burden of LBP, effective treatment and improvement of patient quality of life are particularly critical.

There are many methods for treating LBP clinically, including medication, exercise, manual therapy, physical therapy, dry needling, neural mobilization, cognitive functional therapy, education, etc. (5, 6). However, considering the side effects and adverse reactions of long-term drug use, guidelines suggest against the routine use of opioids for acute LBP and discourage their use for chronic LBP (7). Currently, the clinical practice guidelines of the American College of Physicians recommend non-pharmacological treatments such as superficial heat, massage, acupuncture, or spinal manipulation (8). Silver needle therapy (SNT) is derived from traditional acupuncture, where the silver needles are placed in muscles, tendons, and fascia rather than acupuncture points, and a specialized machine is used to heat the needles to eliminate aseptic inflammation and alleviate pain (9). SNT primarily alleviates pain through three mechanisms: eliminating aseptic inflammation, improving blood circulation, and relieving muscle spasms (10). The pain control mechanism of silver needle therapy is similar to moxibustion, but moxibustion involves burning a cotton ball to generate heat, which does not allow for temperature control. The silver needles are heated by a special device, with the temperature set according to patient feedback. Although SNT has been refined over a long period of development, it still lacks robust clinical evidence to prove its efficacy. Therefore, it is necessary to conduct meta-analysis to evaluate the clinical effectiveness of SNT for pain management in patients with LBP. The results of this meta-analysis will provide evidence for better clinical decision-making and future directions for further clinical trials.

We aim to conduct a meta-analysis and trial sequential analysis (TSA) of randomized clinical trials (RCTs) to assess the clinical efficacy and safety of SNT in the pain management of patients with LBP.

## 2 Materials and methods

### 2.1 Design and registration

This protocol is reported in accordance with the reporting guidelines provided in the Preferred Reporting Items for Systematic Reviews and Meta-analyses Protocols (PRISMA-P) statement. This meta-analysis will be conducted in accordance with the Cochrane Handbook for Systematic Reviews of Intervention, 2nd edition. This protocol has been registered in the PROSPERO database and the registered number is CRD42023466207.

### 2.2 Study selection

#### 2.2.1 Study types

Only RCTs examining the clinical efficacy of SNT for pain management in patients with LBP will be included. There will be no language restrictions. Studies comparing SNT with SNT combined with other analgesic techniques will be excluded if data cannot be used for statistical analysis, if data is incomplete, if data cannot be extracted after contacting the original authors, or if the study is a duplicate publication, such as research published in the form of letters, editorials, conference proceedings, and review summaries.

#### 2.2.2 Participations

Adult participants (age ≥ 18 years) with any LBP condition receiving SNT for pain treatment will be included. There are no restrictions on the participants’ gender, race, body mass index, or the American Society of Anesthesiologists classification.

#### 2.2.3 Interventions/controls

The intervention group will consist of participants who receive SNT alone or in conjunction with any other types of treatment techniques for managing LBP, while the control group will receive any type of treatment techniques other than SNT for managing LBP.

#### 2.2.4 Outcomes

##### 2.2.4.1 Primary outcome

The primary outcome will be the intensity of pain after management through SNT or other treatment techniques. Pain intensity, mainly post-treatment pain intensity, will include assessments using the Visual Analogue Scale (VAS) scores, Numerical Rating Scale (NRS) scores, or other scale scores. If possible, static and dynamic pain intensity after the treatment will also be included.

##### 2.2.4.2 Secondary outcomes

The Oswestry Disability Index is the most commonly used outcome measure to gauge a patient’s permanent functional disability and is considered the gold standard among tools measuring low back function (11). It is composed of 10 items evaluating the severity of a patient’s LBP, self-care ability, and capacity to perform various day-to-day activities, with each question scored from 0 to 5, then summed to derive a total score. Higher scores generally indicate worse conditions (12).

The Japanese Orthopedic Association Back Pain Evaluation Questionnaire is a reliable and sensitive disability measurement method used to determine the functional status of LBP and to assess treatment efficacy. It consists of 25 questions evaluating LBP patients from five different perspectives: pain-related illness, lumbar spine dysfunction, gait disturbance, social life dysfunction, and mental disturbance (13).

Requirement for analgesic drugs, which encapsulates the cumulative use of opioid medication or other pain-relief drugs during and after treatment, including all modes of administration.

Treatment-related adverse reactions, which could occur during or after SNT treatment, such as local bruising, persistent soreness or numbness, fainting sensations, thermal burns, etc.

### 2.3 Electronic bibliographic databases

From the inception of databases until December 2023, published literature in both English and Chinese electronic databases will be searched. English databases will include PubMed, Medline, Cochrane Library, and Embase. Chinese databases will encompass China National Knowledge Infrastructure (CNKI) and Wanfang Databases. Trial registries (ClinicalTrials.gov and WHO International Clinical Trials Registry Platform) will also be reviewed to avoid missing ongoing or unpublished clinical trials. Additionally, reference lists of each study will be scanned to identify studies that may have been missed.

### 2.4 Search strategy

Two reviewers will conduct the search independently, with any disagreements to be resolved through consultation with a third reviewer, if possible. The search strategy will use the following terms: silver needle, thermotherapy, LBP, and RCT. Relevant search terms will also be translated into Chinese for literature research and study identification in Chinese databases. The literature search results will be updated comprehensively before the final publication of the meta-analysis, to avoid missing studies published in the course of preparing the meta-analysis. The detailed search strategy is submitted in Supplementary Table S1.

### 2.5 Selection of studies

**Figure 1:**
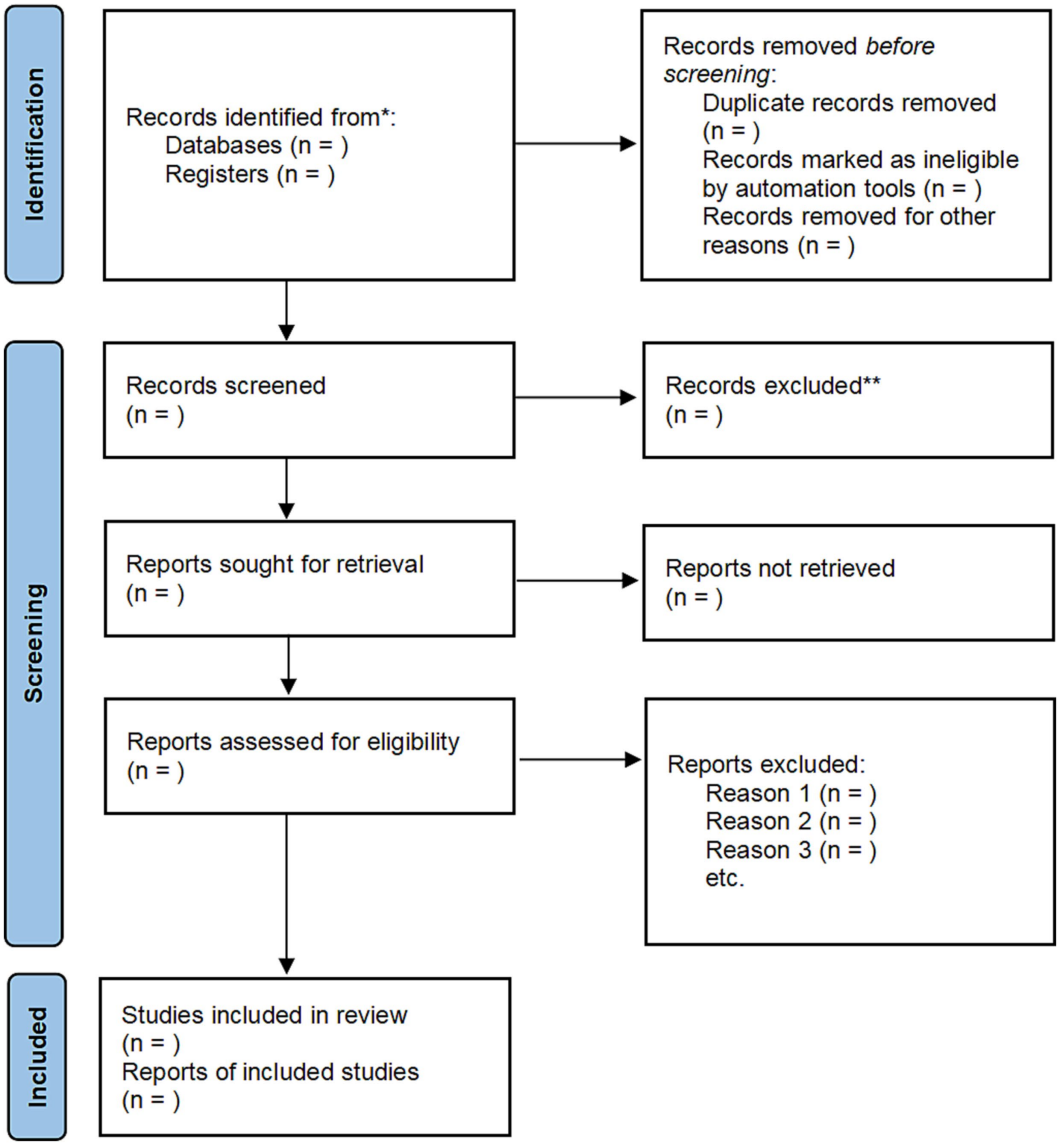
PRISMA 2020 flow diagram.

Two reviewers conducted the literature search independently. The search results were downloaded to Endnote 20 and duplication were excluded. Study screening was conducted in two steps. First, studies that might meet the inclusion criteria were selected based on the title and abstract section of the literature, and then the two authors would identify randomized controlled trials that met the inclusion criteria based on the full text of the studies. If the two reviewer disagree on the selection, the third reviewer will solve disagreements. The study selection process is shown in the PRISMA flowchart Figure 1.

### 2.6 Data extraction

The data extraction form includes demographic data of participants, the degree and location of LBP, the etiology of LBP, inclusion and exclusion criteria, detailed information about the treatment protocol, and any outcomes, including primary, secondary, and exploratory outcomes. Characteristics of the study design will also be recorded, including randomization methods, allocation concealment, blinding (patients, treatment providers, outcome assessors), collection of incomplete outcome data, and statistical analysis and reporting of results. Continuous and dichotomous data will be recorded as *x* ± *S* deviation and percentages or proportions. If any data is unknown or missing, we will contact the original author to clarify the data. If necessary, numerical data from figures will be extracted using Adobe Photoshop (14).

### 2.7 Risk of bias

Two reviewers will independently assess the risk of bias by the Revised Cochrane Risk-of-Bias (RoB2) tool. We will evaluate randomization, deviation from the intervention’s original plan, outcome data missing, measurement of the outcome, and selection of the reported result. We will judge each study as high-risk of bias, some concerns of bias or low-risk of bias. Any disagreements regarding the assessment of risk of bias will be resolved by discussion.

### 2.8 Data synthesis

This review will analyze the data by using Review Manager version 5.4 (Revman 5.4). Because of the potential heterogeneity of the intervention thresholds and intervention methods used, a random effects model will be used. Since both the primary and secondary outcomes are continuous variables and have the same units, we will calculate their mean differences and 95% confidence intervals. If the mean and variance are not reported in a trial, we will estimate the sample mean and standard deviation from the sample size, median, range and/or interquartile range. Meta-analyses will be performed only when two or more included studies reported the same outcome.

Statistical heterogeneity will be measured by the Cochran’s Q and I^2^ statistics, *p* < 0.1 was considered statistically significant for Cochran’s Q, and I^2^ > 75% taken to indicate considerable heterogeneity. If the heterogeneity is significant, we will investigate the source of it and find ways to reduce the heterogeneity.

The results of the meta-analysis will be interpreted according to clinical and statistical significance. A statistically significant reduction in any outcome indicator has some clinical significance. No additional analysis will be conducted in this study. If a quantitative synthesis is not appropriate, this study will only describe and analyze the study results in textual terms

### 2.9 Trial sequential analysis

The required information size (RIS) will be calculated to correct the risks of random errors by TSA using the TSA program V.0.9.5.10 Beta (Copenhagen Trial Unit, Copenhagen, Denmark) (15). TSA program version is available at http://www.ctu.dk/tsa (16). Every outcome will be monitored through RIS, the cumulative Z-curve, and the TSA monitoring boundary to prevent the risk of false-positive (type I error) and false-negative (type II error) results. We will keep a two-sided type I error rate at 5% (alpha boundary), and calculate the required RIS with 80% power, assuming a clinically significant difference of 20% (17).

### 2.10 Subgroup analysis

Subgroup analysis will be conducted to comprehensively interpret the results through analysis of subgroups or subsets wherever possible. If there are enough trials, data from different age groups of subjects, different types of LBP or different locations of LBP, different treatment regimens, and different treatments in control groups will be analyzed independently.

### 2.11 Quality of evidence

Two reviewers will evaluated strength of evidence related to all outcomes using the Grades of Recommendation Assessment, Development and Evaluation (GRADE) method, The quality of effect estimates will be classified as high, moderate, low or very low depending on the risk of bias, consistency, directness, precision and publication bias (18). Data from RCTs are generally considered to be of high quality, but it can be downgraded due to the risk of bias, imprecision, inconsistency, indirectness, or publication bias in the experimental design or implementation.

## 3 Discussion

For many years, LBP has remained a significant public health burden, leading to a substantial amount of work-related disability and healthcare costs (19). It is estimated that between 70 and 85% of the general population will experience at least one episode of LBP at some point in their lives (20). LBP can be categorized into three types based on the duration of the symptoms following an episode. Acute LBP is defined as pain that persists for less than 4 weeks, subacute LBP lasts for 4 to 8 weeks and chronic LBP is characterized by symptoms that persist for more than 8 weeks since onset. There are many treatment methods for LBP, however, the optimal treatment plan has not yet been determined (21). In China, SNT is commonly considered an effective treatment for chronic LBP, a study utilizing SNT to treat chronic nonspecific LBP found that SNT was superior to physical therapy in improving patients’ pain scores, and the therapeutic effect lasted for more than 6 months (22). Furthermore, recent studies have investigated the effects of SNT in treating acute LBP caused by lumbosacral disc degeneration, and have found that SNT can effectively alleviate disability and pain in patients, both in the short and long term (23).

SNT have been developed in China for over 60 years, and are widely used in the treatment of myofascial pain, while fascia plays an important role in LBP (24). Research has found that SNT can reduce the levels of IL-6, IL-8, and TNF-α (25), decrease the expression of neuronal nitric oxide synthase and substance P (26). Earlier numerous scholars have begun to explore 5-HT receptors’ contribution to the regulation of pain. Research has indicated that the ability of these receptors to either amplify or dampen pain signals is tightly linked to the specific receptor types and their action locations (27, 28). Recently scientific inquiries have brought to light 5-HT3 receptors role in the spinal cord’s descending facilitation, a process that may potentially escalate to central sensitization. Lv et al. (29) showed an elevated expression of 5-HT3 receptors in the spinal cords of rats with myofascial pain, pointing to a probable connection between 5-HT3 receptors and myofascial pain related central sensitization. Furthermore, our studies have discovered that administering silver needle thermal therapy can notably reduce spinal 5-HT3 receptors expression in myofascial pain rat models, consequently alleviate pain feeling.

This meta-analysis will summarize the current evidence on the clinical efficacy and safety of SNT in treating patients with LBP. We will examine the analgesic effects, the benefits in reducing disability rates, and the incidence of treatment-related adverse events. The results of this systematic evaluation will aid in clinical decision-making to better treat LBP. The protocol for this meta-analysis was rigorously implemented in accordance with the PRISMA-P guidelines. The strengths of this meta-analysis include: First, a comprehensive literature search of both Chinese and English databases. Secondly, we will conduct multivariate analysis (including subgroup analysis, TSA, sensitivity analysis, etc.) to enhance the quality of evidence. Thirdly, the literature search, data extraction, and assessment of study quality will be independently conducted by at least two review authors according to the guidelines. Any disagreements will be resolved through discussion or consultation with other review authors wherever possible.

Limitations are as follows: First, studies involving LBP of varying locations, etiologies, and durations will be included, leading to potential heterogeneity. Secondly, there is a scarcity of clinical research on silver needle therapy for LBP, hence, the sample size of each included study may be limited and the number of studies with data available for subgroup analysis may be small. Thirdly, studies with high-level evidence, such as well-designed double-blind randomized controlled trials might be limited due to the difficulty in blinding patients.

## Ethics statement

Ethical approval was not required for the study involving humans in accordance with the local legislation and institutional requirements. Written informed consent to participate in this study was not required from the participants or the participants’ legal guardians/next of kin in accordance with the national legislation and the institutional requirements.

## Author contributions

WL: Writing – review & editing, Writing – original draft. XX: Writing – review & editing. RL: Writing – review & editing, Writing – original draft.
