# Supplementary material for: The efficacy of silver needle therapy for treating low back pain: a protocol for meta-analysis of randomized controlled trials
Source: Front Med (Lausanne). 2024 Apr 2;11:1355262. doi: 10.3389/fmed.2024.1355262 (PMC11022203; doi:10.3389/fmed.2024.1355262)
Supplement: Supplementary file 1 [file Data_Sheet_1.docx]

Search strategy of english database as follows:

#1 "low back pain"[MeSH Terms] OR ("low"[All Fields] AND "back"[All Fields] AND "pain"[All Fields]) OR "low back pain"[All Fields] OR ("back pain"[MeSH Terms] OR ("back"[All Fields] AND "pain"[All Fields]) OR "back pain"[All Fields]) OR ("low"[All Fields] AND ("back"[MeSH Terms] OR "back"[All Fields]) AND ("fasciitis"[MeSH Terms] OR "fasciitis"[All Fields] OR "fasciitides"[All Fields])) OR ("low"[All Fields] AND ("back"[MeSH Terms] OR "back"[All Fields]) AND ("myofascial pain syndromes"[MeSH Terms] OR ("myofascial"[All Fields] AND "pain"[All Fields] AND "syndromes"[All Fields]) OR "myofascial pain syndromes"[All Fields] OR ("myofascial"[All Fields] AND "pain"[All Fields] AND "syndrome"[All Fields]) OR "myofascial pain syndrome"[All Fields] OR "fibromyalgia"[MeSH Terms] OR "fibromyalgia"[All Fields]))

#2 (("silver"[MeSH Terms] OR "silver"[All Fields] OR "silvers"[All Fields] OR "silvered"[All Fields]) AND ("needle s"[All Fields] OR "needled"[All Fields] OR "needles"[MeSH Terms] OR "needles"[All Fields] OR "needle"[All Fields] OR "needling"[All Fields] OR "needlings"[All Fields])) OR (("silver"[MeSH Terms] OR "silver"[All Fields] OR "silvers"[All Fields] OR "silvered"[All Fields]) AND ("needle s"[All Fields] OR "needled"[All Fields] OR "needles"[MeSH Terms] OR "needles"[All Fields] OR "needle"[All Fields] OR "needling"[All Fields] OR "needlings"[All Fields]) AND ("thermal"[All Fields] OR "thermalization"[All Fields] OR "thermalize"[All Fields] OR "thermalized"[All Fields] OR "thermalizes"[All Fields] OR "thermalizing"[All Fields] OR "thermally"[All Fields] OR "thermals"[All Fields]) AND ("therapeutics"[MeSH Terms] OR "therapeutics"[All Fields] OR "therapies"[All Fields] OR "therapy"[MeSH Subheading] OR "therapy"[All Fields] OR "therapy s"[All Fields] OR "therapys"[All Fields])) OR (("silver"[MeSH Terms] OR "silver"[All Fields] OR "silvers"[All Fields] OR "silvered"[All Fields]) AND ("needle s"[All Fields] OR "needled"[All Fields] OR "needles"[MeSH Terms] OR "needles"[All Fields] OR "needle"[All Fields] OR "needling"[All Fields] OR "needlings"[All Fields]) AND "Warm"[All Fields] AND ("acupunctural"[All Fields] OR "acupuncture"[MeSH Terms] OR "acupuncture"[All Fields] OR "acupuncture therapy"[MeSH Terms] OR ("acupuncture"[All Fields] AND "therapy"[All Fields]) OR "acupuncture therapy"[All Fields] OR "acupuncture s"[All Fields] OR "acupunctured"[All Fields] OR "acupunctures"[All Fields] OR "acupuncturing"[All Fields])) OR ("Warm"[All Fields] AND ("acupunctural"[All Fields] OR "acupuncture"[MeSH Terms] OR "acupuncture"[All Fields] OR "acupuncture therapy"[MeSH Terms] OR ("acupuncture"[All Fields] AND "therapy"[All Fields]) OR "acupuncture therapy"[All Fields] OR "acupuncture s"[All Fields] OR "acupunctured"[All Fields] OR "acupunctures"[All Fields] OR "acupuncturing"[All Fields])) OR (("silver"[MeSH Terms] OR "silver"[All Fields] OR "silvers"[All Fields] OR "silvered"[All Fields]) AND ("needle s"[All Fields] OR "needled"[All Fields] OR "needles"[MeSH Terms] OR "needles"[All Fields] OR "needle"[All Fields] OR "needling"[All Fields] OR "needlings"[All Fields]) AND ("diathermy"[MeSH Terms] OR "diathermy"[All Fields] OR "diathermies"[All Fields]) AND ("therapy"[MeSH Subheading] OR "therapy"[All Fields] OR "treat"[All Fields] OR "treating"[All Fields] OR "treated"[All Fields] OR "treats"[All Fields])) OR ("Warm"[All Fields] AND ("needle s"[All Fields] OR "needled"[All Fields] OR "needles"[MeSH Terms] OR "needles"[All Fields] OR "needle"[All Fields] OR "needling"[All Fields] OR "needlings"[All Fields]))

#3 "controlled clinical trial"[Publication Type] OR "randomized controlled trial"[Publication Type] OR "randomized"[Title/Abstract] OR "randomized"[Title/Abstract] OR "Placebo"[Title/Abstract] OR "randomly"[Title/Abstract] OR "Clinical trial"[Title]

#4 #1 AND #2 AND #3

Search strategy of chinese database as follows:

(腰[全部字段]or 下背[全部字段]or 脊柱[全部字段]or 椎间盘[全部字段]or 腰椎[全部字段] or 腰痛[全部字段] or 腰肌筋膜[全部字段]or 腰椎关节炎[全部字段]or 骨折[全部字段]or 损伤[全部字段]) and (银质针[全部字段] or 银针[全部字段] or 银针热疗[全部字段]) and (随机[全部字段]or 对照[全部字段])
